# Psychosocial conditions during school-age as determinants of long-term labour market attachment: a study of the Northern Swedish Cohort from the 1980s to the 2020s

**DOI:** 10.1186/s12889-023-17611-6

**Published:** 2024-01-16

**Authors:** Pekka Virtanen, Tapio Nummi, Urban Janlert, Anne Hammarström

**Affiliations:** 1 Tampere University, Tampere, Finland; 2 Umeå University, Umeå, Sweden; 3 Karolinska Institutet, Stockholm, Sweden

**Keywords:** Sweden, Cohort study, Internalised symptoms, Externalised symptoms, Family climate, School connectedness, Labour market, Latent class analysis, Sequence analysis

## Abstract

**Background:**

This study, conducted on a Swedish population cohort, explores how internalized (depressive and functional somatic) and externalized (smoking, drinking, truancy, vandalism, delinquency) mental health symptoms, as well as close interpersonal relations (family climate and school connectedness) reported during adolescence, influence the work-life course up to late midlife.

**Methods:**

We examined repeated measurements of labour market status from age 16 to 56 using sequence analyses. We identified five different labour market attachment (LMA16-56) trajectories, namely ‘strong,’ ‘early intermediate,’ ‘early weak,’ ‘late weak,’ and ‘constantly weak.’ Multinomial logistic regressions were employed to relate each of the nine determinants to the identified trajectories.

**Results:**

When compared to the risk of ‘strong’ LMA16-56, adversity in all conditions, except for vandalism, entailed a higher risk of the ‘constantly weak’ trajectory. Moreover, all conditions, except for functional somatic symptoms, entailed a higher risk of the ‘late weak’ LMA16-56. The risk of the ‘early intermediate’ LMA16-56 was non-significant across all the conditions.

**Conclusions:**

This study contributes to existing knowledge through its novel exploration of labour market attachment and the revelation of the significance of proximal interpersonal relationships in attachment outcomes. Additionally, the study reaffirms the importance of externalizing behaviour, while suggesting that internalized symptoms in adolescence might have a less influential, though not negligible, role. These results underscore the importance of addressing acting out behaviour and nurturing human relationships during compulsory basic education, when the entire age group is still within reach. This approach aims not only to reduce frictions in the school-to-work transition but also to prevent midlife labour market attachment problems that may arise with delayed intervention.

## Background

The reasons for being away from the labour force are manifold, and also within the labour force the stability and intensity of the relationship to work is often suboptimal (for instance fixed-term or part-time employment, temporary agency work, subsistence level entrepreneurship) or there is no relationship at all (i.e. open unemployment and participation in active labour market policy measures). ‘Labour market attachment’ (LMA) [1–3] is a concept that aims to capture individuals’ connectedness to the labour market, encompassing positions both within and outside the labour force. LMA is a correlate of individuals’ life courses as ‘labour market citizens’ [4, 5], which begins upon leaving basic school and concludes at the transition to old-age pension.” The present study uses data on the labour market status of a Swedish population cohort that was followed with six surveys from leaving compulsory school at age 16 to late adulthood at age 56. In this paper we have defined and analysed various LMA trajectories within the cohort across the follow-up period, examining them in relation to the psychosocial conditions at age 16. While childhood adversities have a seminal influence, adolescence is also expected to be another critical period during which poor psychosocial conditions can significantly impact various aspects of the future life course. Research on this topic, however, remains limited [6]. According to the theoretical framework of life course epidemiology [7], socially patterned exposures during adolescence, such as challenges in role exploration, skill development, and especially unsuccessful transitions to post-basic education or work, are believed to have long-term effects on later health and disease risk. Conversely, the reverse effect is also plausible: exposure to poor health during adolescence may labour exert long-term effects on the trajectory of later life, both due to perceived limitations in choices and due to discriminatory social practices.

The focus of the present study is on the psychosocial conditions when teenagers exit from universal compulsory education at age 16 and how these conditions relate to their LMA during the subsequent 40 years. Longitudinal research concerning mental well-being during the school-age years as a determinant of future LMA is scarce. A meta-analysis [6] demonstrates that mental health problems, classified into depression, anxiety, ADHD, and conduct disorder, are more influential than somatic health in predicting labour market outcomes, such as completing secondary school, pursuing post-secondary education, total years of education, employment, or income. However, the authors consider the number of studies included in the meta-analysis to be rather small, especially given that more than half of them are from the USA. More recently, a review [8] focusing on adolescent depression highlights associations with poor educational and labour market outcomes. These reviews also uncover weaknesses in the body of research concerning the outcomes: follow-up times tend to be limited to emerging or early adulthood, and the endpoint status is often indicated cross-sectionally and one-dimensionally.

In addition to individual well-being, it is likely that conditions in various settings surrounding adolescents play a decisive role in shaping their future life course, including their LMA. Referring to Bronfenbrenner’s ecological systems theory, we have defined [9] school and family during adolescence as major arenas within the microsystem. However, we have not found any earlier research on the conditions within the family or school and their impact on future LMA.

The aim of our study is to explore how internalized (depressive and functional somatic) and externalized (smoking, drinking, truancy, vandalism, delinquency) mental health symptoms, as well as close interpersonal relations (family climate and school connectedness) reported during adolescence, influence the work-life course up to late midlife.

## Methods

The studied cohort (the Northern Swedish Cohort, ‘NoSCo’) [10] consists of all pupils who were attending the final grade of compulsory school in an industrial town in Northern Sweden in 1981. Since the baseline survey at age 16, the cohort has been followed by surveys at ages 18, 21, 30, 43, and 56 years. Of the original cohort from 1981 (n = 1083), 1049 were still alive in 2021, of which 942 (89.8%) responded to the questionnaire.

### The determinants

Internalizing symptoms at age 16 were assessed using a six-item depressiveness score and a ten-item functional somatic symptoms score, as detailed earlier [11]. The variables demonstrated acceptable construct validity (Cronbach alphas of 0.65 and 0.70, respectively). The scores were then dichotomized into ‘high’ and ‘low’ categories using the median as the cutoff point.

Dichotomous variables for externalizing symptoms were derived from questions about smoking (yes vs. not currently), drinking (ever been drunk, yes vs. no), truancy (once a month or more vs. a few times per semester or never), vandalism (destroyed property once or more vs. never), and delinquency (reported to the police, yes vs. no).

Proximal relations were assessed using a three-item family climate score and a six-item school connectedness score. Detailed descriptions of the variables, including the items and construct validity (Cronbach alphas of 0.72 for both variables), are provided in a previous article [9]. The scores were subsequently dichotomized into ‘good’ and ‘poor’ categories using the median.

### The outcome: ‘LMA16-56’

The surveys conducted at ages 18, 21, 30, 43, and 56 included questions regarding LMA since the previous survey. For the period of 16–17, data collection involved inquiries about the current labour market status (at school, at work, unemployed, other), the number of weeks employed since completing basic school, the number of weeks unemployed, and school dropouts since finishing basic school.

During the periods 18–21, 22–30, 31–42, and 43–56, LMA was assessed using matrices with time intervals (half-year units) on the x-axis and various status options on the y-axis. These status options were specific to each survey and represented the most relevant and significant statuses during the biography of the cohort and historical context of that time. Respondents who experienced more than one status during a half-year period were instructed to mark all applicable options.

This data was utilized to define age-specific LMA types, categorize them based on LMA strength as ‘strong,’ ‘intermediate,’ or ‘weak’ during each period, and ultimately create the outcome variable ‘LMA16-56,’ which describes the differential development of LMA strength across all periods.

#### LMA during teenage (16–17 years)

Eight LMA types were identified:

1. ‘Education’ (n = 265): Students not unemployed and working less than 5 weeks.
2. ‘Education and employment’ (n = 517): Students working more than 4 weeks and not unemployed.
3. ‘Education and unemployment’ (n = 7): Students unemployed, working less than 5 weeks.
4. ‘Education, employment, and unemployment’ (n = 28): Students both unemployed and working more than 4 weeks.
5. ‘Employment’ (n = 26): Not in school, not unemployed, worked at least one week.
6. ‘Inactive’ (n = 4): Not in school, not unemployed, worked less than five weeks.
7. ‘Unemployment’ (n = 10): Not in school, unemployed, worked less than five weeks.
8. ‘Employment and unemployment’ (n = 78): Not in school, unemployed, worked more than four weeks.

Types 3, 6, 7, and 8 were considered ‘weak’ LMA, type 4 as ‘intermediate,’ and type 5 as ‘strong.’ Types 1 and 2 were classified as ‘strong’ if there were no school dropouts and ‘intermediate’ if one or more school dropouts were present.”

#### LMA during emerging adulthood (18–21 years)

Data from spring 1983 to spring 1986 were analysed using a matrix with 11-status alternatives and 7 time periods. The status alternatives were grouped into ‘education’ (secondary school, university, other), ‘employment’ (full-time, part-time), ‘active labour market policy’ (ALMP), ‘unemployment,’ and ‘other.’

To identify different LMA types, the data, comprising seven repeated binary measurements of five statuses, underwent latent class analysis of multiple responses. This approach extended univariate trajectory analysis [12, 13] to multiple related variables [14, 15]. In our analysis, up to five labour market outcomes were modelled, allowing individuals to occupy more than one position during half-year time units.

A second-degree polynomial on age was considered the most appropriate model for each of the five binary positions, as it allowed for flexibility, including non-linear development within subgroups.

The number of latent classes was determined based on information criteria. Specifically, we used Bayesian Information Criteria (BIC) among the goodness-of-fit statistics provided by Flexmix [16]. There were no significant discrepancies between BIC and other indicators, both for the 18–21 age period and for other periods. BIC pointed to a nine-class solution as the best fit.

**Fig. 1:**
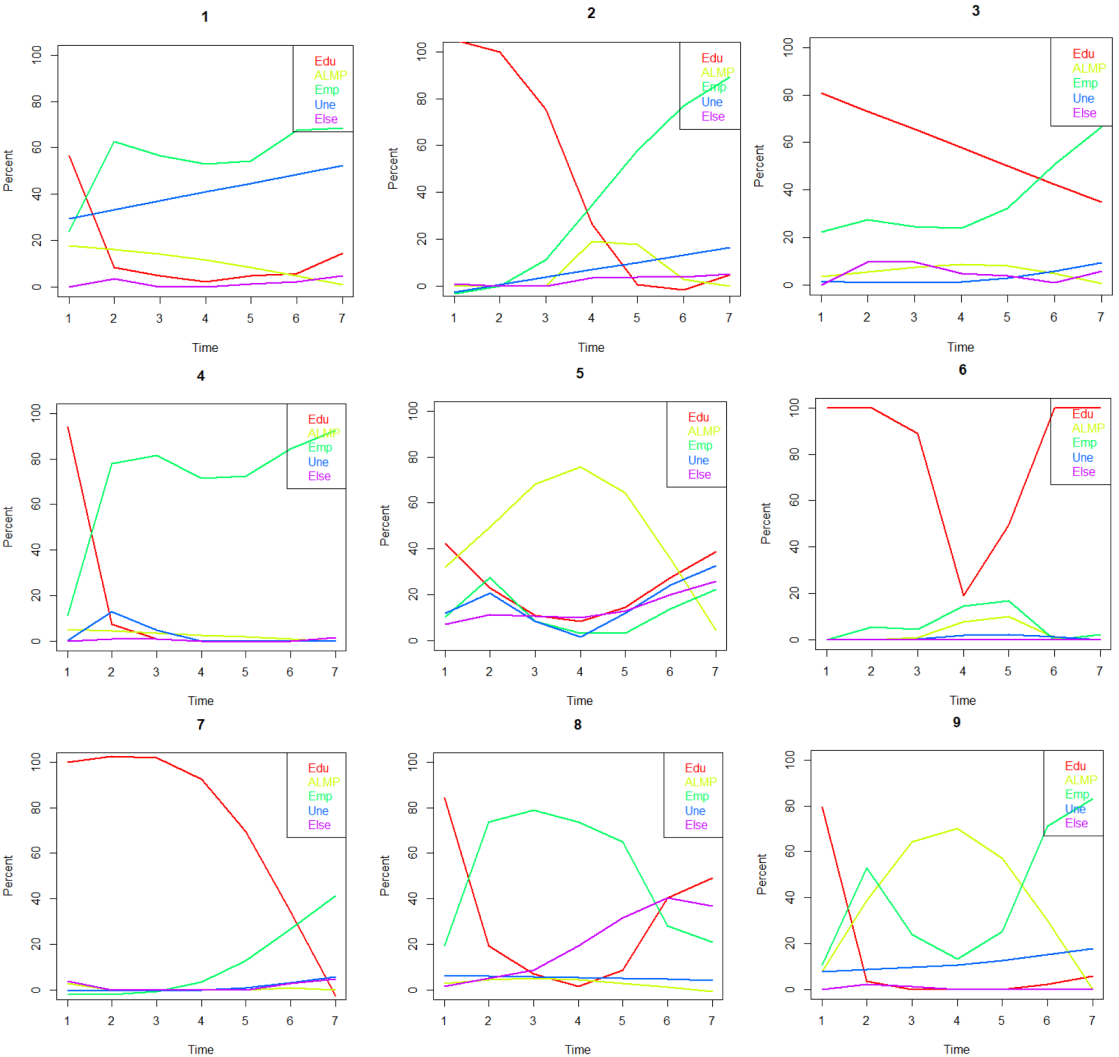
Nine-class typology of labour market attachment of the Northern Swedish Cohort (n = 935) from age 18 to age 21: (1) ‘Unemployment’ (9%), (2) ‘Employment after middle-term education’ (18%), (3) ‘Education and employment’ (12%), (4) ‘Employment after short-term education’ (19%), (5) ‘ALMP and low employment’ (7%), (6) ‘Continuing education’ (9%), (7) ‘Employment after long-term education’ (11%), (8) ‘Transient employment’ (6%), (9) ‘ALMP and high employment (9%). ‘ALMP’: is acronym for ‘enrolled in active labour market policy measure’

The resulting classes, illustrated in Fig. 1, were as follows:

1. ‘Unemployment’: Regular unemployment and low-level employment.
2. ‘Employment after middle-term education’: A relatively smooth transition from education to work around age 20.
3. ‘Education and employment’: Simultaneous study and work.
4. ‘Employment after short-term education’: Transition to work at age 18.
5. ‘ALMP with low employment’: ALMP participation in the middle with a mixture of employment, unemployment, education, and other statuses at the beginning and end of the age window.
6. ‘Continuing education’: Long-term studies interrupted around age 20, likely due to military service.
7. ‘Employment after long-term education’: Initiation of the transition from education to employment around age 21.
8. ‘Transient employment’: Initially transitioning from education to work, but towards the end of the age window, employment declines while education and other statuses increase.
9. ‘ALMP with high employment’: Involves relatively low levels of unemployment and relatively high employment before and after participating in ALMP measures.

Types 1 and 5 were categorized as ‘weak’ LMA, types 8 and 9 as ‘intermediate,’ and types 2, 3, 4, 6, and 7 as ‘strong’ LMA.”

#### LMA during early adulthood (22–30 years)

The 1995 questionnaire featured a matrix with a timeline of 19 half-year periods since fall 1986 and 10 status alternatives. These alternatives were categorized into ‘education’ (university, other), ‘employment’ (including full-time employment, part-time employment > 20 h, and part-time employment < 20 h weekly), ‘unemployment’ (including ALMP measures), ‘parental leave,’ and ‘other’ (including sick leave and other statuses).

Like the 18–21 age group, latent class analysis was used to define LMA types. BIC values consistently indicated a nine-group solution as the best fit.

**Fig. 2:**
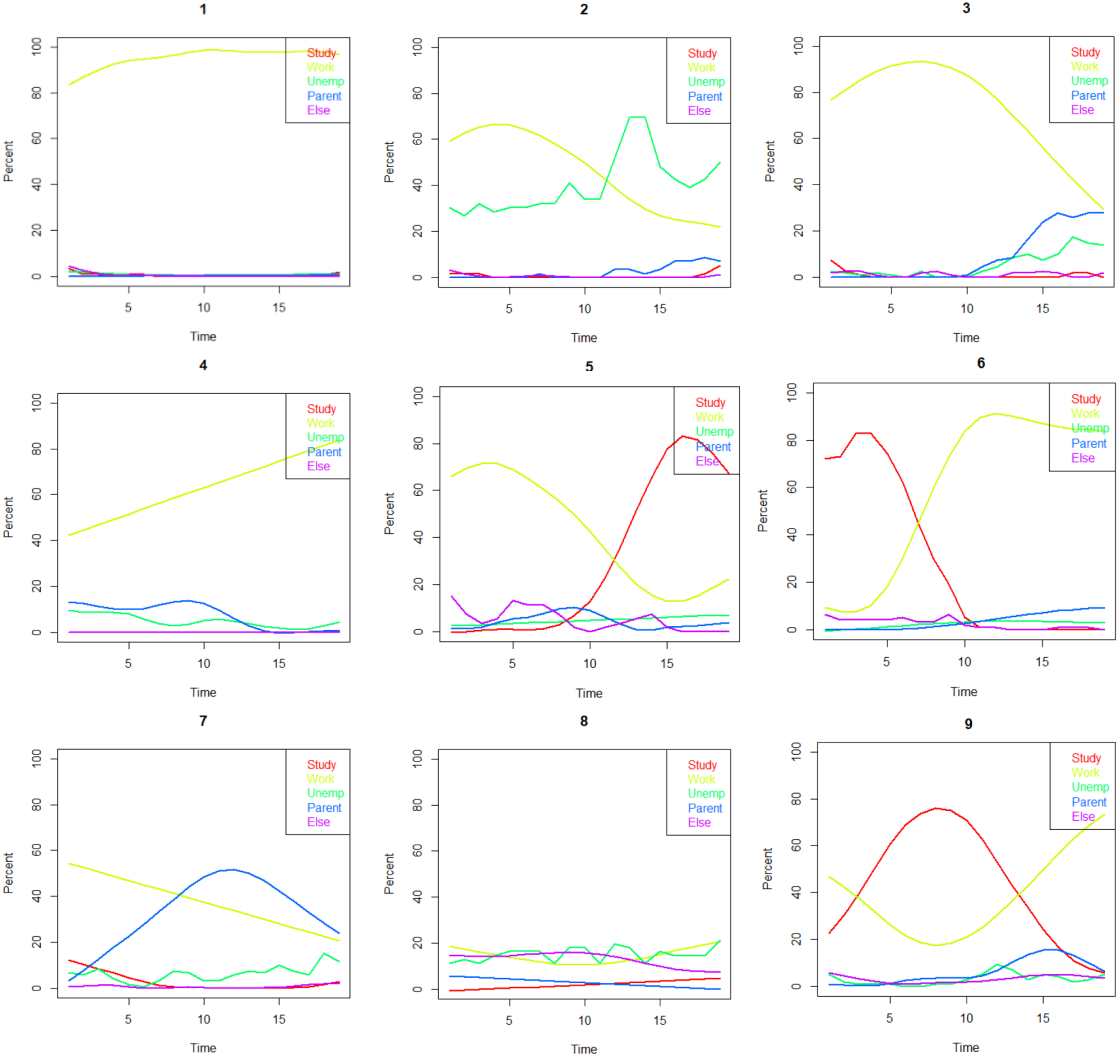
Nine-class typology of labour market attachment of the Northern Swedish Cohort (n = 935) from age 22 to age 30: (1) ‘All-time employment’ (27%), (2) ‘Unemployment’ (6%), (3) ‘Out of employment’ (12%), (4) ‘Into employment’ (10%), (5) ‘From employment to education’ (6%), (6) ‘From education to employment’ (10%), (7) ‘Parent’ (13%), (8) ‘Ambiguous’ (6%), (9) ‘Education and employment’ (10%)

The resulting LMA types (Fig. 2) were as follows:

1. ‘All-time employment’: The largest group, comprising approximately one in four participants.
2. ‘Unemployment’: Participants who consistently shift between unemployment and employment.
3. ‘Out of employment’: Participants who, by the end of this age range, have mostly transitioned into parental leave or, to a lesser extent, unemployment.
4. ‘Into employment’: Individuals whose employment starts relatively low in the beginning (possibly due to parental leaves or unemployment) but increases significantly by the end.
5. ‘From employment to education’: A relatively small group, likely influenced by the economic recession, who pursued various studies in the early 1990s.
6. ‘From education to employment’: Participants who transitioned into the workforce around age 25.
7. ‘Parent’: Participants who took parental leaves during this age range, with a slight decline in employment and a slight increase in unemployment.
8. ‘Ambiguous’: A relatively small group characterized by LMA that cannot be categorized using the five status alternatives.
9. ‘Education and employment’: Those who simultaneously pursued education and employment and eventually transitioned entirely into the workforce by age 30.

Types 2 and 8 were categorized as ‘weak’ LMA, types 3, 5, and 7 as ‘intermediate,’ and types 1, 4, 6, and 9 as ‘strong’ LMA.”

#### LMA during early middle age (31–42 years)

**Fig. 3:**
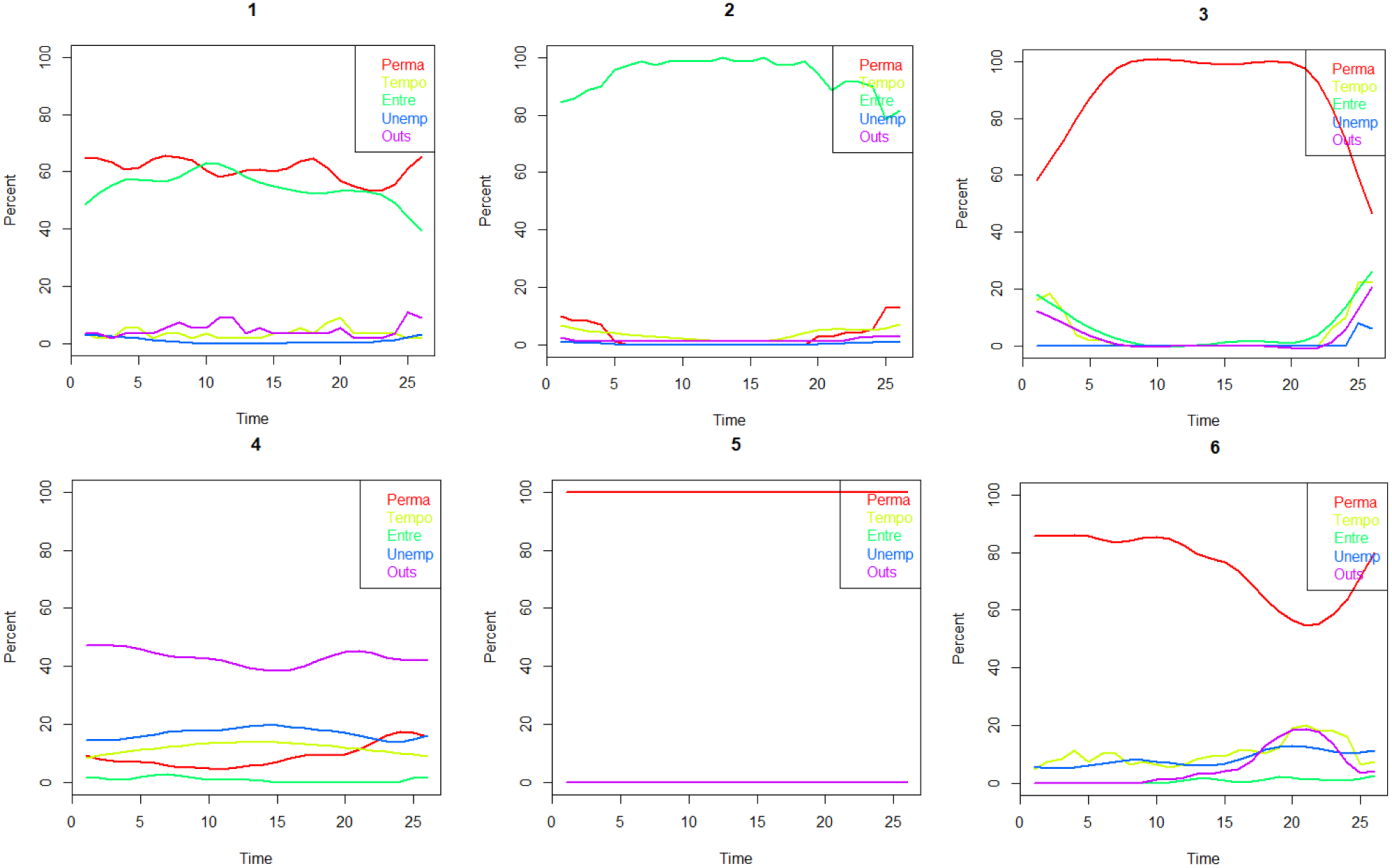
Six-class typology of labour market attachment of the Northern Swedish Cohort (n = 935) from age 43 to age 56: (1) ‘Permanent and entrepreneur’ (6%), (2) ‘Entrepreneur’ (7%), (3) ‘Permanent 1’ (5%), (4) ‘Outside’ (11%), (5) ‘Permanent 2’ (59%), (6) ‘Sagging permanent’ (11%)

The matrix of the 2007 questionnaire consisted of a timeline spanning 24 half-years and included 11 statuses. For latent class analysis, these statuses were condensed into five categories: ‘employment with a permanent contract,’ ‘entrepreneur,’ ‘employment with a temporary contract’ (encompassing project work, substitutes, probationers, on-demand jobs, seasonal jobs, and other fixed-term jobs), ‘unemployment’ (including participation in ALMP measures), and ‘other’ (out of the labour force). A seven-type solution (Fig. 3) was found to be the most informative.

**Fig. 4:**
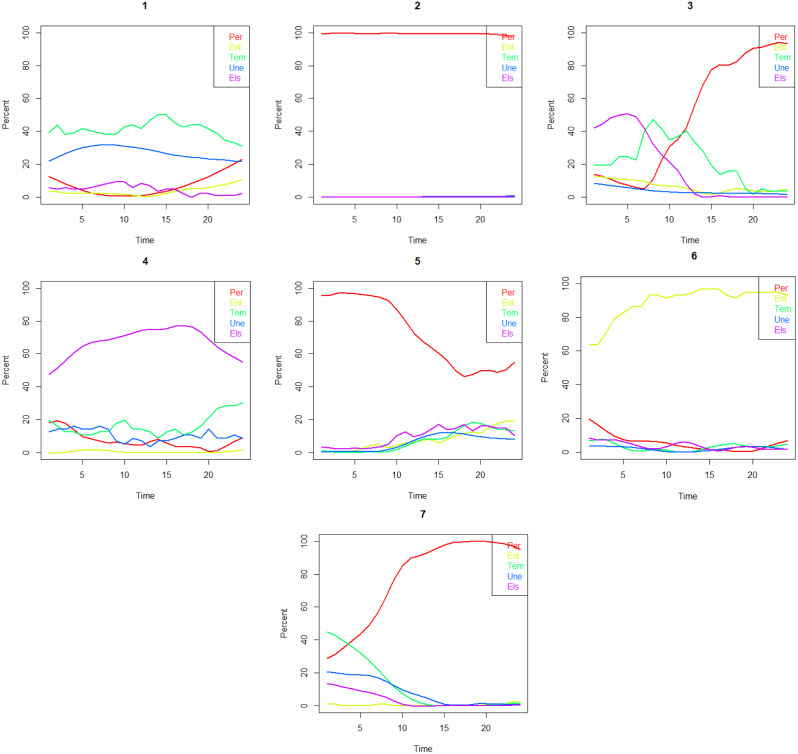
Seven-class typology of labour market attachment of the Northern Swedish Cohort (n = 935) from age 13 to age 42: (1) ‘Temporary employment and unemployment’ (9%), (2) ‘All-time permanent’ (48%), (3) ‘Into permanent 1’ (6%), (4) ‘Else’ (6%), (5) ‘Declining permanent’ (15%), (6) ‘Entrepreneur’ (6%), (7) ‘Into permanent 2’ (10%)

The resulting latent classes (Fig. 4) were named as follows:

1. ‘Temporary employment and unemployment’: Participants who oscillate between various temporary contracts and periods of unemployment.
2. ‘All-time permanent employment’: Nearly half of the cohort maintains permanent contracts throughout the age range.
3. ‘Into permanent 1’: Participants who secure permanent employment after initially occupying other positions.
4. ‘Else’: Individuals whose predominant status remains ‘else’ throughout the period.
5. ‘Declining permanent’: Those who tend to change their initially permanent status to other statuses by the end of the time window.
6. ‘Entrepreneurs’: Participants predominantly engaged in entrepreneurship throughout the period.
7. ‘Into permanent 2’: Individuals who, unlike those in ‘Into permanent 1,’ obtain permanent employment mostly after initially experiencing temporary employment or unemployment.

Types 1 and 4 were categorized as ‘weak’, type 5 as ‘intermediate’ and types 2, 3, 6 and 7 ‘strong’ LMA.

#### LMA during late middle age (43–56 years)

**Fig. 5:**
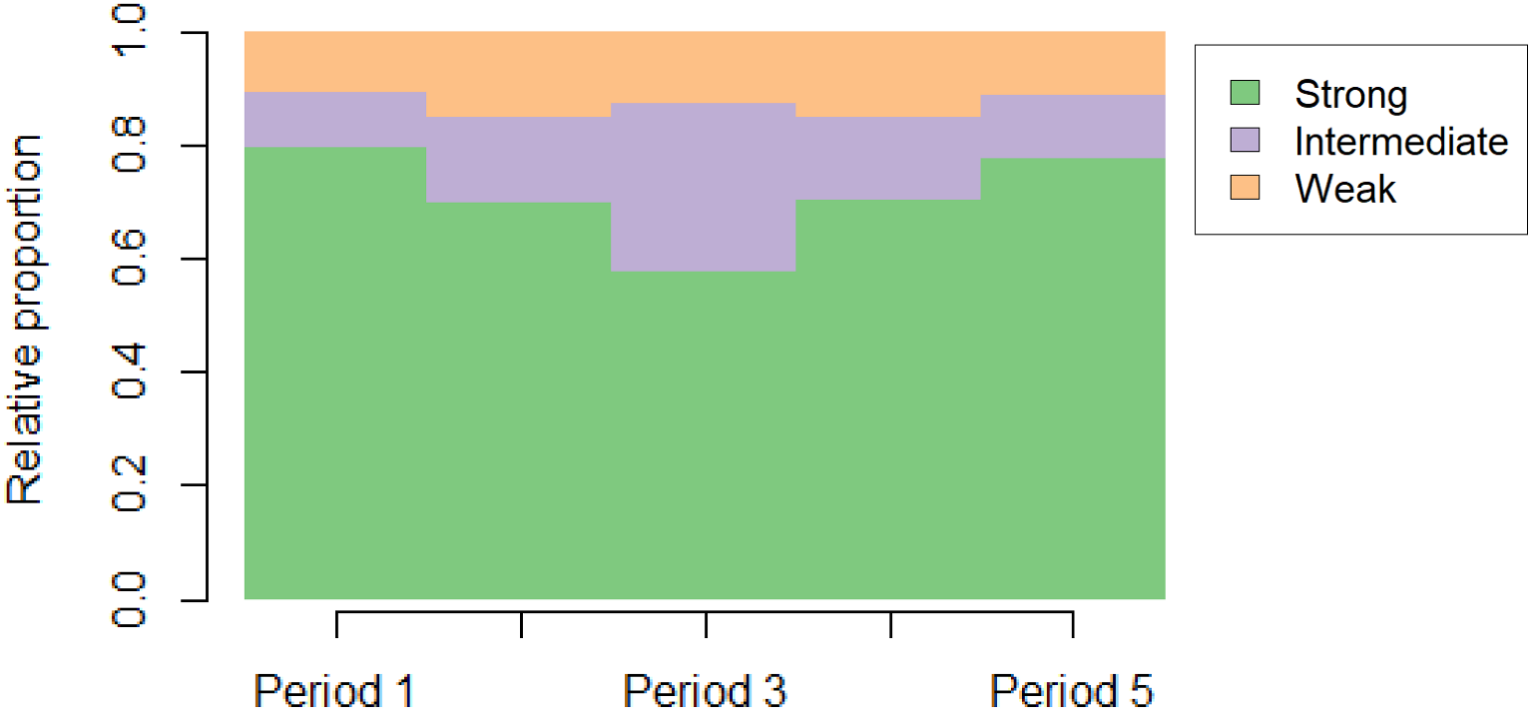
Labour market attachment of the Northern Swedish Cohort (n = 935) from age 16 to age 56. Period 1: 16–17 y, period 2: 18–21 y, period 3: 22–30 y, period 4: 31–42 y, period 5: 43–56 y

The 2021 questionnaire featured a matrix with a timeline spanning 26 half-year periods and included the following status alternatives: permanent employment, entrepreneur, temporary employment, unemployment (including ALMP), and being outside the labour force. In the latent class analysis, a six-group solution (Fig. 5) was determined to be the most suitable.

The resulting latent classes (Fig. 3) were defined as follows:

1. ‘Permanent and entrepreneur’: Participants who evidently work simultaneously as entrepreneurs and as employees.
2. ‘Entrepreneur’: Those who exclusively engage in entrepreneurship.
3. ‘Permanent 1’: Participants whose permanent contracts increase at the beginning and decrease by the end of the age window.
4. ‘Outside’: Individuals who are predominantly outside the labor force but may also experience periods of unemployment or temporary employment to some extent.
5. ‘Permanent 2’: A large group of participants with permanent employment throughout the age window.
6. ‘Sagging permanent’: Participants with a decline in permanent employment and an increase in temporary employment and periods outside the labor force before the end of the age window.

Type 4 was categorized as representing weak LMA, type 5 as intermediate, and types 1, 2, 3, and 6 as strong LMA.

Figure 5 summarizes LMA during different age periods according to strength. The proportion of cohort members with strong LMA is highest, at 80%, during ages 16–17, and nearly as high, at 78%, during ages 43–56, with the lowest figure at 58% during ages 21–30. The proportions of weak LMA range from 10 to 15%.

**Fig. 6:**
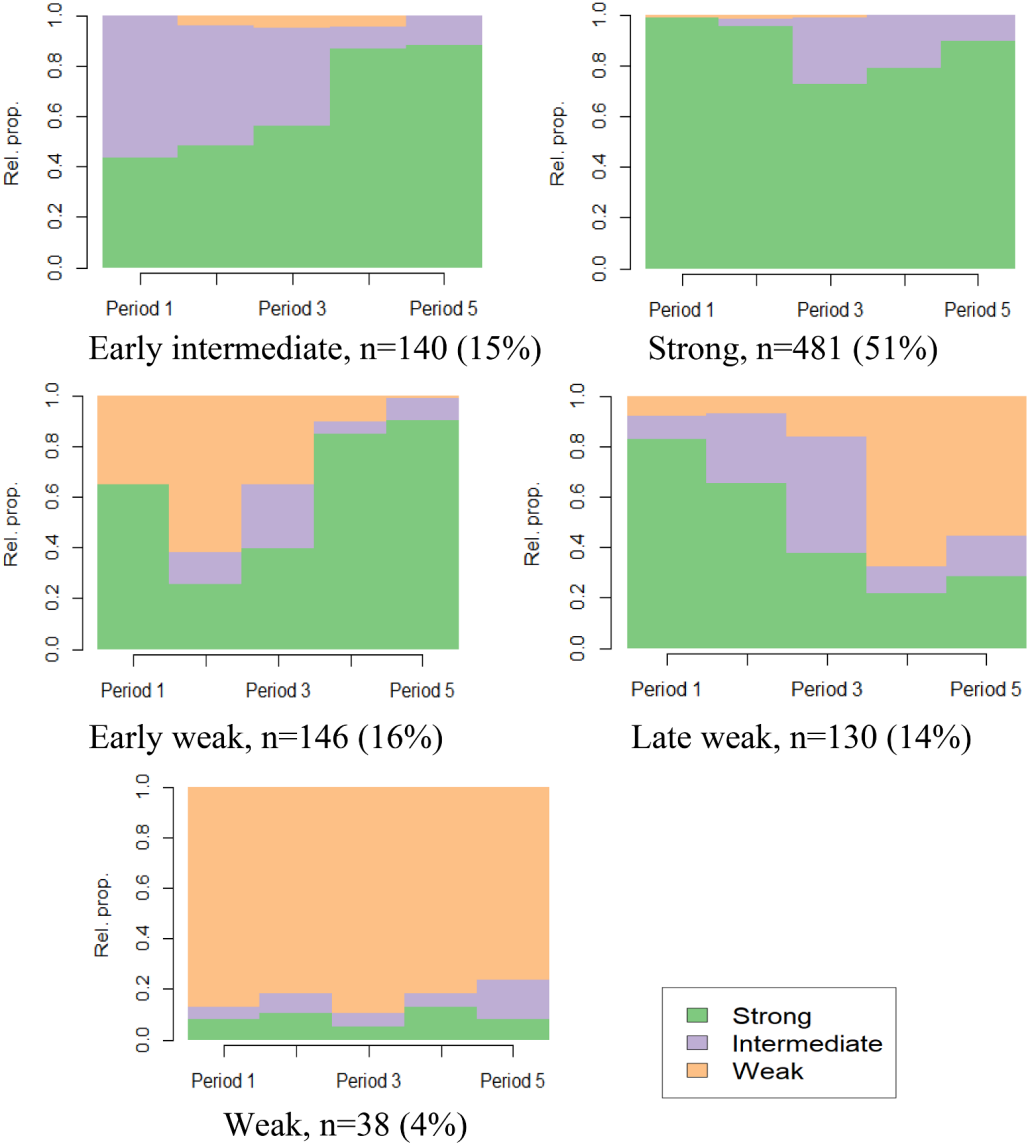
Division of the Northern Swedish Cohort according to labour market attachment from age 16 to age 56 (five-group sequence analysis)

Finally, we studied variations in the strength of LMA variables across the five age periods using sequence analysis. This analysis was conducted with the TraMineR package in R software [17]. The procedure involved forming a distance matrix using the optimal matching method and then performing hierarchical clustering using Ward’s method. The five-group solution (Fig. 6) was considered the best, considering both interpretation and the proportion of participants assigned to the sequence groups. The obtained variable was named LMA16-56. The sequence group with predominantly strong LMA throughout the follow-up, comprising 51% of the cohort, ‘strong’, and the group (15%) that transitions from intermediate to strong by age ‘early intermediate’. The sequence analysis also identified an ‘early weak’ group (16%), a ‘late weak’ group (14%), and a smaller ‘constantly weak’ group (4%).

Associations of the determinants with LMA16-56 were studied using multinomial logistic regression analyses. In addition to analysing each binary psychosocial condition separately, depressiveness was combined with school connectedness into a four-class variable (no-no, no-yes, yes-no, yes-yes). Gender and parents’ socioeconomic status (no/one/both working-class parents) were included in the models as potential confounders. The regression analyses were conducted using IBM SPSS Statistics for Windows, version 27 (IBM Corp., Armonk, N.Y., USA).

## Results

To address the second and the third hypotheses of the study, we analysed the importance of the above defined nine psychosocial conditions for the LMA, spanning from age 16 to age 56. As demonstrated in Fig. 6, this LMA16-56 variable categorizes the attachment as ‘strong’, ‘early intermediate’, ‘early weak’, ‘late weak’ or ‘constantly weak’.

Descriptive statistics (Table 1) show that women are overrepresented in the ‘late weak’ group and, to some extent, in the ‘early intermediate’ group. Most of those in the ‘strong’ LMA16-56 category have parents from a high social class, while the opposite trend is observed in the ‘early weak’ and particularly in the ‘constantly weak’ group. In terms of internalizing symptoms, low scores tend to be less common in the ‘late weak’ and ‘constantly weak’ groups. Regarding externalizing symptoms, the prevalence of ‘no/seldom’ responses for all five indicators is highest in the ‘strong’ group and lowest in the ‘constantly weak’ group. A similar pattern of results is observed with proximal relations as the determinant.

**Table 1:** Labour market attachment trajectories of the Northern Swedish Cohort from age 16 to 56 according to the psychosocial conditions at age 16

Conditions	Strong n = 481	Early interm. n = 140	Early weak n = 146	Late weak n = 130	Constant weak n = 38	All n = 935
**Backgrounds**						
Women	44%	54%	49%	62%	45%	48%
*Family SES*						
– Blue collar	29%	38%	46%	39%	68%	36%
– Mixed collar	37%	34%	35%	32%	16%	35%
– White collar	34%	28%	19%	29%	16%	29%
**Internalizing, low symptom score**						
Depression	49%	46%	38%	33%	24%	42%
Functional somatic	60%	62%	61%	50%	42%	41%
**Externalizing, seldom/no**						
Smoking	68%	58%	46%	50%	26%	59%
Ever drunk	53%	45%	35%	40%	22%	46%
Truancy	83%	78%	65%	72%	29%	75%
Vandalism	67%	64%	60%	62%	58%	64%
Delinquency	90%	87%	77%	81%	62%	85%
**Proximal relations, good**						
Family climate	61%	51%	40%	38%	26%	52%
School connectedness	58%	58%	39%	41%	37%	51%

The results of multivariate multinomial regression analyses, with ‘strong’ LMA16-56 and good condition as references, are presented in Table 2. In the internalizing category, depression increases the risk of belonging to the ‘late weak’ and ‘constantly weak’ groups, while a high functional somatic symptom score is marginally associated with an increased risk for the ‘constantly weak’ group. Regarding externalizing symptoms, vandalism is specifically associated with an increased risk for the ‘late weak’ group, while the other indicators pose risks for all types of weak groups. Notably, truancy is associated with a ten-fold increase in the risk of belonging to the ‘constantly weak’ LMA16-56 group. Additionally, there are significant associations between poor family climate and school connectedness with weak LMA groups.

**Table 2:** Internalized and externalized symptoms and proximal social relationships at age 16 as determinants of labour market attachment trajectories from 16 to 56 years of age

Determinant category Variables	Labour market attachment trajectory			
	Early intermediate RRR (95% CI)^a^	Early weak RRR (95% CI)^a^	Late weak RRR (95% CI)^a^	Constantly weak RRR (95% CI)^a^
**Internalizing: high vs. low**				
Depression	1.01 (0.69–1.50)	1.46 (0.98–2.17)	**1.69 (1.10–2.57)**	**3.13 (1.41–6.94)**
Functional somatic symptoms	0.85 (0.57–1.25)	0.93 (0.63–1.37)	1.34 (0.90–1.99)	**1.98 (1.00-3.90)**
**Externalizing: yes vs. no**				
Smoking	1.42 (0.96–2.11)	**2.46 (1.66–3.62)**	**1.91 (1.28–2.86)**	**6.06 (2.83-13.0)**
Ever drunk	1.36 (0.93–1.99)	**2.13 (1.44–3.14)**	**1.67 (1.12–2.49)**	**4.29 (1.91–9.65)**
Truancy	1.35 (0.84–2.15)	**2.43 (1.60–3.70)**	**1.82 (1.14–2.88)**	**10.3 (4.86–21.7)**
Vandalism	1.30 (0.85-2.00)	1.40 (0.92–2.13)	**1.76 (1.13–2.75)**	1.40 (0.67–2.91)
Delinquency	1.42 (0.79–2.57)	**2.66 (1.60–4.43)**	**2.67 (1.53–4.64)**	**5.28 (2.44–11.4)**
**Proximal relations: poor vs. good**				
Family climate	1.41 (0.96–2.06)	**2.14 (1.46–3.14)**	**2.33 (1.56–3.49)**	**4.06 (1.91–8.62)**
School connectedness	1.01 (0.69–1.48)	**2.16 (1.47–3.16)**	**1.99 (1.33–2.96)**	**2.45 (1.23–4.88)**

Relative risk ratios (RRR) from multinomial logistic regression models. **Bold** font indicates a statistically significant association

^a^Reference trajectory ‘strong’, adjusted for gender and parent socioeconomic status

We also examined the associations between different combinations of depressiveness and school connectedness with LMA16-56, as these factors are a subject of concern and public discussion among adolescents (Table 3). When looking at the percentages, differences between the ‘strong’ and ‘early intermediate’ groups are minimal, while the three weak groups exhibit similar patterns. Regression analyses, adjusted for gender and socioeconomic background, indicate that compared to the ‘strong’ group, the relative risks of belonging to the other groups are non-significant when depression is present without poor school connectedness. Conversely, poor school connectedness without depression predicts membership in the ‘early weak’ and ‘late weak’ LMA16-56 groups. When both conditions coexist, the risk is non-significant only for the ‘early intermediate’ group.

**Table 3:** Labour market attachment from age 16 to 56 according to the combinations of high score of depressive symptoms (‘depression’) with poor school connectedness (‘school’) at age 16

	Strong n = 481	Early intermediate n = 140	Early weak n = 146	Late weak n = 130	Constantly weak n = 38
None, n = 270	36%	30%	19%	18%	16%
Depression only, n = 215	23%	28%	21%	23%	21%
School only, n = 135	13%	16%	19%	15%	8%
Both, n = 314	29%	26%	41%	45%	55%
None	ref	ref	ref	ref	ref
Depression only	ref	1.31 (0.79–2.18)	1.63 (0.90–2.92)	1.72 (0.94–3.15)	2.05 (0.68–6.21)
School only	ref	1.44 (0.80–2.61)	**2.81 (1.53–5.15)**	**2.30 (1.17–4.54)**	1.34 (0.32–5.57)
Both	ref	1.00 (0.60–1.65)	**2.64 (1.57–4.44)**	**2.69 (1.56–4.62)**	**4.50 (1.73–11.7)**

Relative risk ratios from multinomial logistic regression analyses, adjusted for gender and socioeconomic background. **Bold** font indicates a statistically significant association

## Discussion

Using a novel approach to analyse questionnaire data on labour market status over forty years, this study identified five groups based on different levels of labour market attachment (‘LMA16-56’). These groups include the optimal ‘strong’ attachment group, as well as the suboptimal ‘early intermediate,’ ‘early weak,’ ‘late weak,’ and ‘constantly weak’ groups.

Nine potential early determinants of non-strong LMA16-56 were examined. None of the determinants were associated with the ‘early intermediate’ group, while six determinants were linked to a high risk of ‘early weak’ LMA16-56. For the ‘late weak’ LMA16-56 group, all determinants except functional somatic symptoms were associated with a high risk, and for the ‘constantly weak’ LMA16-56 group, all conditions except vandalism were associated with a high risk.

Among the confounders, parents’ socioeconomic status deserves particular attention. Participants from blue-collar families are notably more likely to belong to the ‘constantly weak’ LMA16-56 group compared to offspring from white-collar families. Similar disparities are evident in the other non-strong LMA groups. This observation underscores the profound influence of socioeconomic background on labour market inequality.

In earlier studies on this topic, the outcome has typically been a specific educational or employment-related status until a certain age. In contrast, the present study assesses all relevant and major labour market statuses over a 40-year period following the completion of basic education. Nevertheless, it is valuable to interpret earlier research findings in light of the present results. Regarding depression, while the current study found that the risk of depressiveness did not reach statistical significance for the ‘early weak’ LMA16-56 group, it did reveal a higher risk for the ‘late weak’ and ‘constantly weak’ LMA16-56 groups, supporting previous evidence of this association, particularly in the long term [8]. These results are somewhat inconsistent with an earlier study within the NoSCo [18], which indicated that the risk of depressive symptoms at age 16 leading to an unstable trajectory from age 18 to 42 was not significant. These discrepancies may arise from differences in the methods used to analyse LMA, variations in statistical power (as men and women were studied separately), and variations in follow-up times. As the influence of psychosomatic symptoms on LMA has not been previously explored, the present study can be considered a reference for future research. Nevertheless, the non-significant associations support the notion that the impact of somatic complaints, even though related to mental health, on future labour market outcomes is relatively minor compared to the impact of mental health-related issues.

In our study, we considered ‘everyday misconduct,’ which has often been examined as a mediator [19] or confounder [20] when studying the effects of long-term conditions and attention/conduct problems on labor market attachment (LMA). Interestingly, McLeod et al. [21], in their research on academic achievement, found results consistent with ours. They observed that smoking and delinquency were predictive of poor achievement, aligning with our findings. However, the relatively low risk associated with ‘early onset’ drinking in their study contrasts with our results. Our study also highlights the significance of truancy. In addition to its association with poor educational attainment and NEET (Not in Employment, Education or Training) status in the short term [19], it carries a notable risk for weak LMA in later life.

Despite the lack of earlier studies on the proximal relations as determinants of LMA, the associations of poor family climate and school connectedness with weak LMA could be expected on the basis of existing life course studies [22, 23]. Conceptualizing them as proximal relations instead of adverse experiences means that the teenager is seen as an active partner in the social relationships rather than just an exposed victim. We interpret the results as support to the critical importance of the interaction between the induvial and the micro-level settings [24]. There is an evident need for corresponding studies with determinants that capture more comprehensively proximal conditions in childhood as well as during emerging adulthood.

Overall, poor psychosocial conditions certainly often coincide. Table 3 illustrates the occurrence of depression and poor school connectedness in relation to LMA16-56. These two conditions are often considered as major problems affecting success at school and subsequently in the labour market. While the combination of a high depressive symptom score and poor connectedness is more prevalent than a low score with good connectedness, there are significant numbers of individuals experiencing each condition separately. Enough cases in the four subgroups allowed for multinomial logistic regression analysis. This analysis indicates that the prognosis for LMA is not poor as long as the depressed pupil remains committed to school. However, in the case of a poorly connected pupil without lowered mood, there are significant risks of weak attachment both in early stages and later on.

Measuring determinants as teenagers transition from compulsory schooling to becoming independent labour market participants excludes reverse causation, meaning the influence of LMA on the determinants. Like the sensitive period model of life course epidemiology, this study assumes that psychosocial conditions persist over time in individuals, albeit potentially changing its importance. Depression, for instance, starts to be a distinctive entity at puberty [25], and although adolescence-onset depression has some specific features [26], having once occurred depressiveness tends to recur across early adulthood and middle age [27, 28]. This has also been shown in NoSCo [29]. The results of this study suggest just a moderate influence of internalised mental symptoms on success in the labour market. On part of the externalizing symptoms, assuming that they, or in the case of truancy and vandalism corresponding behaviour, remain stable across the life course, the ‘early weak’ and the ‘late weak’ LMA16-56 tell that considerable part of the cohort succeeds in strengthening the LMA despite unfavourable lifestyle. Regarding externalizing symptoms, assuming that they remain stable across the life course—or in the case of truancy corresponding behaviour—the ‘early weak’ and ‘late weak’ LMA16-56 categories indicate that a significant portion of the cohort manages to improve their labour market attachment despite an unfavourable lifestyle. Regarding poor family climate and school connectedness, the exposures cannot continue in their actual contexts during the subsequent life course. However, it is possible that these factors predispose individuals to similar problems in emerging adulthood and middle age, ultimately weakening their labour market attachment.

The findings with individual-level (i.e. externalizing and internalizing symptoms) determinants parallel the results of an earlier study following up the NoSCo for 14 years, from age 16 to 30, which identified bivariate associations between early smoking and future socioeconomic status in both males and females. Similarly, an association of psychological symptoms was observed in men only, and it was either small or non-existent except for the relationship of overweight to working-class position in women [30]. On the other hand, a paper from age 30 to 42 demonstrated health-related selection into unemployment which seems to take place through difficult re-employment rather than through risk of losing the job [31]. Like the earlier papers, the current study prompts a discussion regarding whether the associations between poor conditions and weak LMA result from individual factors or labour market factors, such as discriminatory practices. Of course, the screening processes for post-primary and higher education can be interpreted as either macro-level or population-level discrimination or as individual failures. In the context of employment, the impact of mental health and health behavior might be more pronounced during recruitment, while the influence of proximal adult relationships becomes more prominent during downsizing.

Within the research field of social inequalities in health, Patrik West [32] a long time ago argued for redirection of research on health selection as a question of discrimination, where people with poor health are at risk of discrimination on the labour market and thus risk an increased tendency to drift down the social scale. The hypothesis implies that employers select employees so that those with poor health become discriminated.

Reverse causality must be considered when analysing work-related exposures during life. While the findings appear to support the concept of health selection contributing to inequalities in the labor market, it does not negate the fact that LMA contributes to the development of health inequalities during the life course [33].

The study aligns with the objectives of our on-going research programme, which seeks to integrate the theory of life course epidemiology with Bronfenbrenner’s social influence theory, as introduced in the 1970s Bronfenbrenner’s “The Ecology of Human Development: Experiments by Nature and Design” [34] and elaborated upon in recent papers [9, 35]. Viewed through the lens of Bronfenbrenner’s theory, this study serves as a test of the significance of proximal conditions in adolescence for human development, exemplified in this case by the importance of close relationships during adolescence for future success in the labour market.

Furthermore, within the realm of life course research, this study illustrates the complexity of the work life course and serves as a foundational exploration of labor market attachment as a theoretical concept.

There are inherent methodological challenges in the measurement of mental health problems in longitudinal research. There is constant development in definitions, taxonomies and demands concerning the properties of mental health measurements. We have tested the properties of the mental health measures used in when NoSCo was initiated according to the standards of today and conclude that composite measures of mental health problems can be constructed from single items which are more than 30 years old [11]. These measures seem to have the same factorial structure and internal consistency across a significant part of the life course.

Maintaining a consistent set of status alternatives in the time-status matrices of the NoSCo surveys over the decades proved challenging, as the relevance and prevalence of different statuses varied over time and with age. Due to this, the procedure of obtaining a LMA measure common to all five age periods consisted of, first, latent class analyses of the period specific data into qualitatively different LMA types and, second, categorizing each type into a class of strong, intermediate or weak LMA. This quantitative variable was then used for conducting sequence analysis to categorize the cohort based on their labour market attachment development up to age 56. The multiple response latent class analysis method employed here has been utilized in only a limited number of studies [36, 37]. The method has the advantage of utilising fully the information, as it enables capturing more than one status during the time unit. For instance, one may study and work simultaneously, be on parental leave with a valid permanent contract, and shuttle between unemployment and employment. This empirical procedure allows for the examination of work-related life courses throughout the working age of a population sample, regardless of whether individuals are temporarily or permanently within or outside the labour force. Importantly, this approach aligns with the theoretically defined latent phenomenon, which is conceptualized as labour market attachment.

The groups identified through latent class and sequence analysis are not exclusive categories. In latent class analysis, they are based on the highest posterior probability of group membership, while in sequence analysis, they result from seeking a reasonable number of meaningful and distinct groups. In our study, the posterior probabilities were notably high, indicating successful group assignments. The sequence analysis, which spans five episodes with a three-alternative variable, and the chosen five-cluster solution do involve some inherent uncertainty. However, these groups effectively represent real-life LMA and serve as a reasonable outcome variable for the multinomial regression analyses.

We emphasize that our methodology involves two subjective assessment elements. Firstly, it involves categorizing a latent class as strong, intermediate, or weak attachment. Secondly, the determination of the number of clusters for the sequence analysis is based on study-specific criteria, such as the perceived real-life correspondence of trajectories and the need to maintain sufficiently large trajectory groups for further analyses.

The definition of the LMA concept, as well as the data collected in the NoSCo study, are based on administrative labour market statuses. Therefore, the latent classes and categories of the LMA16-56 variable also need to be labelled using corresponding concepts. The contents behind these labels are explained with brief texts, but its important to note that attributing subjective qualities like job insecurity, job commitment, employability, etc., is beyond the scope of the present study.

Due to the population-level sample with a high participation rate, there are only minor, if any, biases related to psychosocial conditions or LMA. The cohort has proven to be comparable to the country as a whole in terms of socio-demographic factors, health status, and health behaviour [10]. On the other hand, the study is based on data from a specific cohort originating in a Northern Swedish industrial town and followed over a particular historical period. This limits application of the results to today’s adolescents, as well as their generalizability internationally. Replication studies are needed, potentially utilizing register data to mitigate recall bias inherent in self-reported retrospective labour market status data, are essential. Replication studies with more established and modern mental health variables is also warranted, particularly given the cohort-specific nature of the score variables– as discussed in [9].

The LMA variables hold promise for future longitudinal studies within NoSCo, particul–rly when examining health outcomes. Moreover, this exploration of LMA can be viewed as a step toward a deeper understanding of the work-life course, encompassing the age-related status transitions and variations of the status. Advancements are needed both in the collection and analysis of empirical data and in the development of theory, especially in response to historically changing roles of individuals as ‘labour market citizens’.Employing the LMA concept in a longitudinal setting implies consideration of macro-level labor markets regulations such as the rate of unemployment. Using the LMA concept in a longitudinal setting implies consideration of macro-level labour markets. The lack of contextualisation could be one reason why life-course epidemiology is much rarer in research on work life. As a criticism of the fragmented research in the field, Amick et al. [38] conceptualised also work as life-course processes depending on place and time, with focus on labour market transitions and trajectories that various groups face during the life-course. They emphasize the need to understand the role of various contextual levels in shaping labour markets and health trajectories to identify relevant policies and interventions. The LMA variables hold promise for future longitudinal studies within NoSCo, particularly when examining health outcomes. Moreover, this exploration of LMA can be viewed as a step toward a deeper understanding of the work-life course, [38] encompassing the age-related status transitions and variations of the status. Advancements are needed both in the collection and analysis of empirical data and in the development of theory, especially in response to historically changing roles of individuals as ‘labour market citizens’.

Finally, the results of this study can be considered within the framework of the NEET concept (Not in Employment, Education, or Training, which has been established to refer to young people facing challenges in LMA. It appears that NEET predicts a consistently weak LMA trajectory quite infrequently (4%), whereas among those assigned to the ‘early weak’ trajectory (16%), it often remains a transient episode. Nonetheless, these results underscore the importance of addressing disruptive behaviour and nurturing human relationships during universal basic education, while the entire age group is still accessible. This effort is not only aimed at reducing frictions in the school-to-work transition but also at mitigating LMA issues that manifest with delay in middle age. Furthermore, school health care should strive to identify and support depressed pupils, especially those who exhibit weak commitment to attending school. This state of affairs is probably similar nowadays, even though a higher proportion of pupils continue to and finalize secondary high school, thus reducing the number of early NEETs. On the other hand, the higher unemployment rate compared to the 1980s tends to increase the number of NEETs.

## Conclusions

In terms of their influence on LMA, externalizing behavior and the quality of close interpersonal relationships during adolescence are more significant than internalized mental health symptoms. This finding underscores the importance of efforts to reduce disruptive behavior and nurture positive human relationships during compulsory basic education when the entire age group is still accessible. These efforts aim not only to minimize challenges in the school-to-work transition but also to prevent LMA issues that may emerge later in middle age.

## Data Availability

The datasets analysed during the current study are not publicly available due to the Swedish Act (SFS 2003:460) on ethical review of research involving humans, which does not permit sensitive data on humans to be freely shared. Selections of the datasets are available after ethical permission and after request to the PI Anne Hammarström (anne.hammarstrom@ki.se).

## Abbreviations

LMA: labour market attachment
LMA16-56: five-group classification of the labour market attachment from age 16 to age 56 by strength
NoSCo: Northern Swedish Cohort
NEET: Not in Employment Education or Training

## References

[1] Laux R. Measuring labour market attachment using the Labour Force Survey, Labour Market trends 1997 (October), London: HMSO.

[2] Little A (2007). Inactivity and labour market attachment in Britain. Scott J Political Econ.

[3] Virtanen P, Lipiäinen L, Hammarström A, Janlert U, Saloniemi A, Nummi T (2011). Tracks of labour market attachment in early middle age: a trajectory analysis over 12 years. Adv Life Course Res.

[4] Suikkanen A, Viinamäki L, Ferrie E, Marmot M, Griffiths J (1999). New dimensions of labour market citizenship. Labour market changes and job insecurity: a challenge for Social Welfare and health promotion.

[5] Andersen J, Jensen P, Andersen J, Jensen P (2002). Citizenship, changing labour markets and welfare policies: an introduction. Changing labour markets, welfare policies and citizenship.

[6] Hale D, Bevilacqua L, Viner R (2015). Adolescent health and adult education and employment: a systematic review. Pediatrics.

[7] Kuh D, Ben-Shlomo Y, Lynch J, Hallqvist J, Power C (2003). Life course epidemiology. J Epidemiol Community Health.

[8] Clayborne Z, Varin M, Colman I (2019). Systematic review and meta-analysis: adolescent depression and long-term psychosocial outcomes. J Am Acad Child Adolesc Psychiatry.

[9] Gunnarsdottir H, Hensing G, Hammarström A (2021). Poor school connectedness and adulthood depressiveness: longitudinal theory-driven study from the Northern Sweden Cohort. Eur J Pub Health.

[10] Hammarström A, Janlert U (2012). Cohort Profile: the Northern Swedish Cohort. Int J Epidemiol.

[11] Hammarström A, Westerlund H, Kirves K, Nygren K, Virtanen P, Hägglöf B (2016). Addressing challenges of validity and internal consistency of mental health measures in a 27-year longitudinal cohort study– the Northern Swedish Cohort study. BMC Med Res Methodol.

[12] Nagin D (1999). Analyzing developmental trajectories: semiparametric, group-based approach. Psychol Methods.

[13] Nagin D (2005). Group-based XXXodelling of development.

[14] Nagin D, Jones B, Lima Passos V, Tremblay R. 2016. Group-based multi-trajectory modelling. Statistical Methods in Medical Research 2016;27:2015–23. 10.1177/0962280216673085 29846144

[15] Girard L, Tremblay R, Nagin D, Côté S (2018). Development of aggression subtypes from childhood to adolescence: a group-based multi-trajectory modelling perspective. J Abnorm Child Psychol.

[16] Leisch F, FlexMix (2004). A general framework for finite mixture models and latent class regression in R. J Stat Softw.

[17] Gabadinho A, Ritschard G, Studer M, Muller N. Mining sequence data in R with the TraMineR package: a user’s guide. Univ Geneva, 2010. (http://mephisto.unige.ch/traminer).

[18] Landstedt E, Brydsten A, Hammarström A, Virtanen P, Almquist Y. The role of social position and depressive symptoms in adolescence for life-course trajectories of education and work: a cohort study. BMC Public Health (2016) 16. 10.1186/s12889-016-3820-4 PMC5116205 27863527

[19] Hale D, Viner R (2018). How adolescent health influences education and employment: investigating longitudinal associations and mechanisms. J Epidemiol Community Health.

[20] Evensen M, Lyngstad T, Melkevik O, Mykletun A (2016). The role of internalizing and externalizing problems in adolescence for adult Educational Attainment: evidence from sibling comparisons using data from the Young HUNT Study. Eur Sociol Rev.

[21] McLeod J, Uemura R, Rohrman S (2012). Adolescent mental health, behavior problems, and academic achievement. J Health Soc Behav.

[22] Metzler M, Merrick M, Klevens J, Ports K, Ford D (2017). Adverse childhood experiences and life opportunities: shifting the narrative. Child Youth Serv Rev.

[23] Bellis M, Hughes K, Ford K, Ramos Rodriguez G, Sethi D, Passmore J (2019). Life course health consequences and associated annual costs of adverse childhood experiences across Europe and North America: a systematic review and meta-analysis. Lancet Public Health.

[24] Bronfenbrenner U, Morris PA. The bioecological model of human development. In: Damon W, Lerner RM, editors. Handbook of Child Psychology. Vol. 1: Theoretical Models of Human Development, 6th edn. Hoboken, NJ: John Wiley & Sons, Inc., 2006; 793–828.

[25] Maughan B, Collishaw S, Stringaris A (2013). Depression in childhood and adolescence. Can Acad Child Adolesc Psychiatry.

[26] Shanahan L, Copeland W, Costello E, Angold A (2011). Child-, adolescent- and young adult-onset depressions: Differential risk factors in development?. Psychol Med.

[27] Pine D, Cohen E, Cohen P, Brook J (1999). Adolescent depressive symptoms as predictors of adult depression: moodiness or mood disorder?. Am J Psychiatry.

[28] Thapar A, Collishaw S, Pine D, Thapar A (2012). Depression in adolescence. Lancet.

[29] Lallukka T, Mekuria G, Nummi T, Virtanen P, Virtanen M, Hammarström A (2019). Co- occurrence of depressive, anxiety, and somatic symptoms: trajectories from adolescence to midlife using group-based joint trajectory analysis. BMC Psychiatry.

[30] Hammarström A, Janlert U (2005). Health selection in a 14-year follow-up study– a question of gendered discrimination?. Soc Sci Med.

[31] Virtanen P, Janlert U, Hammarström A (2013). Health status and health behaviour as predictors of the occurrence of unemployment and prolonged unemployment. Public Health.

[32] West P (1991). Rethinking the health selection explanation for health inequalities. Soc Sci Med.

[33] Benach J, Vives A, Amable M (2014). Precarious employment: understanding an emerging social determinant of health. Annu Rev Public Health.

[34] Bronfenbrenner U (1979). The Ecology of Human Development: experiments by Nature and Design.

[35] Nyberg A, Rajaleid K, Westerlund H, Hammarström A (2019). Does social and professional establishment at age 30 mediate the association between school connectedness and family climate at age 16 and mental health symptoms at age 43?. J Affect Disord.

[36] Ek E, Ala-Mursula L, Velázquez R, Tolvanen A, Salmela-Aro K (2021). Employment trajectories until midlife associate with early social role investments and current work-related well-being. Adv Life Course Res.

[37] Saloniemi A, Salonen J, Nummi T, Virtanen P (2021). The diversity of transitions during early adulthood in the Finnish labour market. J Youth Stud.

[38] Amick et al. 2017 (Amick BC Labor markets and health: an integrated life-course perspective. Scand J Work Environ H 2016;42:346– 53. 10.5271/sjweh.3567 27158797

